# Predicting maternal healthcare seeking behaviour in Afghanistan: exploring sociodemographic factors and women’s knowledge of severity of illness

**DOI:** 10.1186/s12884-023-05750-y

**Published:** 2023-08-02

**Authors:** Essa Tawfiq, Mohammad Daud Azimi, Aeraj Feroz, Ahmad Shakir Hadad, Mohammad Samim Soroush, Massoma Jafari, Marzia Salam Yaftali, Sayed Ataullah Saeedzai

**Affiliations:** 1 The Kirby Institute, UNSW Sydney, Sydney, Australia; 2 Formerly the Ministry of Public Health, Kabul, Afghanistan; 3 Management Sciences for Health (MSH), Kabul, Afghanistan; 4 Emory University, Atlanta, USA; 5 McMaster University, Ontario, Canada; 6 Ministry of Public Health, Kabul, Afghanistan

**Keywords:** Antenatal, ANC, Postnatal, PNC, Deliveries, Childbirth, Healthcare seeking, Illness severity

## Abstract

**Background:**

Little is known whether women’s knowledge of perceived severity of illness and sociodemographic characteristics of women influence healthcare seeking behavior for maternal health services in Afghanistan. The aim of this study was to address this knowledge gap.

**Methods:**

Data were used from the Afghanistan Health Survey 2018. Women’s knowledge in terms of danger signs or symptoms during pregnancy was assessed. The signs or symptoms were bleeding, swelling of the body, headache, fever, or any other danger sign or symptom (e.g., high blood pressure). A categorical variable of knowledge score was created. The outcome variables were defined as ≥ 4 ANC vs. 0–3 ANC; ≥ 4 PNC vs. 0–3 PNC visits; institutional vs. non-institutional deliveries. A multivariable generalized linear model (GLM) was used.

**Results:**

Data were used from 9,190 ever-married women, aged 13–49 years, who gave birth in the past two years. It was found that 56%, 22% and 2% of women sought healthcare for institutional delivery, ≥ 4 ANC, ≥ 4 PNC visits, respectively, and that women’s knowledge is a strong predictor of healthcare seeking [odds ratio (OR)1.77(1.54–2.05), 2.28(1.99–2.61), and 2.78 (2.34–3.32) on knowledge of 1, 2, and 3–5 signs or symptoms, respectively, in women with ≥ 4 ANC visits when compared with women who knew none of the signs or symptoms. In women with ≥ 4 PNC visits, it was 1.80(1.12–2.90), 2.22(1.42–3.48), and 3.33(2.00–5.54), respectively. In women with institutional deliveries, it was 1.49(1.32–1.68), 2.02(1.78–2.28), and 2.34(1.95–2.79), respectively. Other strong predictors were women’s education level, multiparity, residential areas (urban vs. rural), socioeconomic status, access to mass media (radio, TV, the internet), access of women to health workers for birth, and decision-making for women where to deliver. However, age of women was not a strong predictor.

**Conclusion:**

Our findings suggest that pregnant women’s healthcare seeking behaviour is influenced by women’s knowledge of danger signs and symptoms during pregnancy, women’s education, socioeconomic status, access to media, husband’s, in-laws’ and relatives’ decisions, residential area, multiparity, and access to health workers. The findings have implications for promoting safe motherhood and childbirth practices through improving women’s knowledge, education, and social status.

**Supplementary Information:**

The online version contains supplementary material available at 10.1186/s12884-023-05750-y.

## Background

Maternal mortality ratio (MMR) and neonatal mortality ratio (NMR) are important indicators of the overall health status of women and newborns. MMR and NMR are still very high in many Low- and Middle-Income Countries (LMICs) [1–3], and with current progress it seems difficult, if not impossible, to achieve the Sustainable Development Goals (SDGs) to end preventable maternal, under-5, and newborn deaths due to modifiable risk factors by the year 2030 [1–3]. These modifiable risk factors could be avoided by increasing access of pregnant women to healthcare services, including antenatal care and postnatal care [4, 5].

Progress towards achieving SDGs on maternal and child health has been hindered in the conflict and post-conflict affected countries. According to the Fragile States Index, in 2020 nine countries (from highest to lowest fragile) were: Yemen, Somalia, South Sudan, the Syrian Arab Republic, the Democratic Republic of Congo, the Central African Republic, Chad, Sudan, and Afghanistan, with their MMRs ranging from 30 (the Syrian Arab Republic) to 1223 (South Sudan) [2]. Outside the sub-Saharan African countries, Haiti (MMR 350) and Afghanistan (MMR 620) had a very high MMR in 2020 [2]. Over the past two decades, MMR has gradually declined in Afghanistan from 1450 deaths in 2000, to 954 deaths in 2010, and to 638 deaths per 100,000 live births in 2017 [6]. Despite this, maternal mortality in Afghanistan is one of the worst globally and among its neighboring countries. For instance, in 2017, the MMR in Pakistan was 140, in Uzbekistan was 29, in Tajikistan was 17, in Iran was 16, and in Turkmenistan was 7 deaths per 100,000 live births [6]. With the shift of political power to the Taliban in August 2021, diseases and deaths among women are expected to rise in Afghanistan due to disruption and reductions in donor funding for the health system, increasing poverty and unemployment, and continued violations of basic human and women’s rights [7].

Women in LMICs often suffer from serious pregnancy-related health conditions that may lead to death. Nearly half a million women die annually as a consequence of complications during pregnancy, childbirth or postpartum period [6]. Pregnancy and childbirth are important events in the life span of a woman, and play a crucial role in the wellbeing of women and their unborn or newborn children [5]. Maternal healthcare, particularly antenatal care and postnatal care, as a continuum of care is vital in assuring the wellbeing of women and their unborn or newborn children, and can greatly contribute to the reduction of maternal morbidity and mortality [8]. Antenatal care (ANC) is described as the care provided by skilled health workers to pregnant women as well as young women in order to ensure the best health conditions for both mother and baby during pregnancy [5]. Risk identification, prevention, and management of pregnancy-related or concurrent diseases, as well as health education and promotion are all elements of ANC. In light of the above, 2016 WHO recommendation on ANC for a positive pregnancy experience modified the minimum number of ANC from 4 to 8 visits, with the first visit commencing within the first 12 weeks of pregnancy [5]. ANC reduces maternal morbidity and mortality both directly and indirectly, through the early detection and management of pregnancy-related complications as well as the identification of women who are at high risk of experiencing complications during labour and delivery, and ensuring that they are referred to the proper level of care [9]. WHO defines the postnatal period as the first six weeks after delivery, which is a crucial time for both a mother and her newborn child [10]. Serious complications may occur in this period which could lead to the death of the mother and her newborn baby [10]. To overcome these risks, WHO recommends 4 PNC visits, with the first visit during the first 24 h, the second visit in 48–72 h, the third visit in 7–14 days, and the fourth visit at six weeks after delivery [10].

Poor healthcare seeking during pregnancy, childbirth, and postpartum period is a contributor to unfavorable pregnancy outcomes [11]. A recent study that analyzed data from 36 Demographic Health Surveys from sub-Saharan African region found that proportion of women initiating healthcare seeking for ANC services during the first 12 weeks of pregnancy was low, ranging from 14.5% in Mozambique to 68.6% in Liberia [12]. Another recent study that used data from 54 DHS and Multiple Indicator Cluster Surveys (MICS) since 2012, reported that initiation of ANC contacts and coverage of ANC visits was still low, with significant variations between countries [13]. Coverage of at least 4 ANC visits during a woman’s pregnancy is still low in LMICs [14–16], and utilization of content-quality ANC component among pregnant women is even lower than the coverage of initiating ANC visits in the first 12 weeks of pregnancy [16–18]. Early postnatal care, defined as at least one PNC check-up for a mother or her newborn by a healthcare provider within the first week after childbirth, and PNC follow-up visits is still low [19–22]. Seeking healthcare, as a preventative strategy, is an ideal way to increase utilization of maternal and child health care, and reduce the risk of pregnancy-related complications for the mother and her unborn or newborn child [5]. Sociodemographic factors such as occupation, multiparity, education, maternal age, and distance to health facilities may affect healthcare seeking behavior of women [23, 24]. The use of reproductive health services by women is related to their education level and that of their husbands [25]. Women’s awareness of the availability and provision of maternal healthcare at the health facilities is a significant predictor of service utilization [26]. Women’s knowledge of pregnancy complications, and education status are significant determinants of healthcare seeking for maternal health services [27]. Women’s perceived necessity to seek maternal healthcare, women’s decision to give birth in a primary, secondary, or tertiary hospital, and a history of pregnancy complications are all strong predictors of healthcare seeking behavior in pregnant women [28].

Several studies in Afghanistan have examined utilization of maternal healthcare and the associated influential factors in recent years [29–39]. Of these, one study found that sociodemographic and cultural (woman’s role in making the decision to seek PNC service) factors were associated with the utilization of PNC visits [29]. Three studies reported that sociodemographic and cultural factors were associated with institutional deliveries or skilled birth attendance (SBA) deliveries [30, 31, 35]. One of the three studies reported that women who had at least four ANC visits were more likely to give birth in a health facility or with SBA than those who had none, and women from higher socioeconomic status used institutional deliveries more than women from lower socioeconomic status [30]. The second study revealed that a history of complications in previous births, the husband’s education, and ANC visits were associated with institutional deliveries [31]. Another of the three aforementioned studies indicated that socioeconomic status and decisions made for women on where to deliver were correlated with SBA deliveries [35]. It was more likely that a woman would use SBA delivery services when the place to give birth was decided by the husband or other relatives (e.g., father-in-law, mother-in-law) than by the woman herself [35]. One of the studies from Afghanistan examined the relationships between non-use of ANC visits and non-institutional deliveries [33]. It was found that the probability of non-institutional deliveries was greater in women with no ANC visit compared to women with at least 4 ANC visits [33]. Four studies from Afghanistan examined the effects of sociodemographic and cultural factors on the utilization of ANC services [34, 36–38]. Among the four studies, one reported that women’s education, women’s intention for pregnancy, and place of residence were influential on the use of ANC services (at least 4 visits) [34]. Another study found that woman’s age, education of woman and husband, area of residence, wealth, and husband decision for healthcare use were associated with ANC service utilization [36]. The third study showed that women's lack of knowledge about the proper timing for ANC visits, their unplanned previous pregnancy, and their unattendance for ANC visits in the past were correlated with late start of first ANC visit (> 12 weeks pregnancy).[37]. In the fourth study, which examined the relationships between male attendance at ANC visits with their expectant wives and the utilization of maternal healthcare, it was found that it was more likely that pregnant women who were accompanied by their husbands would utilize adequate ANC services, begin ANC visits during first trimester, have institutional delivery, and use PNC service compared to women who were not accompanied by their husbands during ANC visits [38]. While most of the studies from Afghanistan used quantitative methods, two of them applied qualitative methods and investigated barriers and obstacles related to the unitization of maternal health services [32, 39].

Of the two studies, one reported that money to pay for services seemed to be the most important barrier to accessing institutional delivery. The second most cited barrier was unavailability of transportation means, followed by family restrictions and cultural constraints to accessing institutional delivery [32]. The second study uncovered the following barriers to accessing institutional delivery: a lack of knowledge on the importance of ANC services among the women and their families, financial difficulties, transportation problems, and dissatisfaction with the attitudes of staff in health facilities [39]. Another qualitative study from Afghanistan found that the respondents women mentioned about the stigma associated with using health facilities for birth, inability to afford the travel cost to health facilities, shame of delivering outside home in the presence of midwives, and women’s lack of decision-making power where to give birth [40].

Considering the literature on the determinants of women’s healthcare seeking behavior for maternal healthcare, little is known about the effect of women’s knowledge of perceived severity of illness on healthcare seeking for pregnancy-related health services. Moreover, recent studies from Afghanistan used data from small samples which may have limited the generalizability of their findings to the national level [31, 34, 37]. In this study, we examined whether women’s knowledge of perceived severity of illness and sociodemographic characteristics of women predict women’s healthcare seeking behavior for at least 4 ANC, 4 PNC visits, and institutional delivery services.

## Methods

### Study design and data source

We used secondary data from the Afghanistan Health Survey 2018 (AHS 2018). Data were collected between March 2018 and August 2018 from 23,460 randomly selected households in the 34 provinces [41]. The AHS 2018 employed a two-stage stratified sampling strategy. Each province was stratified by urban and rural areas. In the first stage within each stratum, residential listing was carried out in the 1,020 randomly selected clusters by visiting each of the clusters to prepare a list of occupied residential households. In the second stage, the list of occupied households was used as the sampling frame, and 23 households from each cluster were randomly chosen using a systematic sampling approach. The surveyors were given the final list of selected households to do the survey. To minimize the risk of selection bias, no substitution in the list of selected households was permitted.

In this study we used data from ever-married women, aged 13–49 years, who gave birth within the past two years of the survey. In the selected households, women were interviewed by trained surveyors, using a questionnaire on seeking healthcare for women and children, and another questionnaire on mortality and verbal autopsy as relevant. The head of household was interviewed, using the questionnaire that included information on the household members' demographic, health, and socioeconomic status. After the data were collected by trained surveyors, the collected data in the survey forms were brought to the Kabul office where data entry and verification started. The collected survey forms were checked, edited, coded, and the data were entered twice by two separate data entry operators on two separate times. A mechanism of consistency check in data entry was set up in the software in which the data were entered. In case of inconsistency, the survey form in question was reviewed again and the data edited and re-entered [41]. To ensure quality of data, daily tracking of survey teams, regular field supervision of the teams, and post-monitoring visits of a sample of the households already surveyed were conducted [41].

### Statistical analysis

There were three binary outcome variables; seeking healthcare for at least 4 ANC, at least 4 PNC visits, and institutional deliveries. The outcome variables were coded as “ ≥ 4 ANC visits vs. 0 – 3 ANC visits”, “ ≥ 4 PNC visits vs. 0 – 3 PNC visits”, and “institutional delivery vs. non-institutional delivery”. An institutional delivery was defined as a childbirth that took place in a health facility. The explanatory variables were women’s knowledge of perceived severity of illness related to pregnancy and sociodemographic characteristics of women. The variable on women’s knowledge of perceived severity of illness had four categories: women with no knowledge of danger signs or symptoms, women who knew 1 sign or symptom, women who knew 2 signs or symptoms, and women who knew 3–5 signs or symptoms. During the interview, women were asked the question “Can you name the signs or symptoms that indicate the need for you to seek urgent care”, with the response options of (i) fever, (ii) bleeding, (iii) swelling of the body, (iv) headache, and (v) others (specify). The sociodemographic characteristics were age of woman (13–29 years, 30–39 years, and 40–49 years), woman’s education level [no formal education, primary education (1–6 years of schooling), intermediate education (7–9 years of schooling), secondary education (10–12 years of schooling), higher education], socioeconomic status (quintile, from poorest to richest), place of residence (urban vs. rural), multiparity (women with a history of one childbirth vs. women with a history of ≥ 2 childbirths), decision where the woman to deliver (decision by herself, decision by husband, decision by her mother or mother- or father-in-law, decision by relatives or friends), last delivery was conducted by a (medical doctor, nurse, midwife, non-medical person), access to radio (women who did not listen to radio, women who listened to radio almost every day, women who listened to radio at least once a week), access to TV (women who did not watch TV, women who watched TV almost every day, women who watched TV at least once a week), access to internet (women who did not use the internet to consult information, women who consulted information on the internet almost every day, women who consulted information on the internet at least once a week). For variables on access to radio, access to TV, and access to the internet, we coded the following women as part of those women who did not have access to these media. These women were those who had access to these media less than once a week.

The variable on socioeconomic status was created using the principal component analysis, using household ownership of assets, household use of amenities of life, and construction materials used for the household buildings. A multivariable generalized linear model (GLM) with binary outcome was specified.

$$Y_{ij}=\beta_0+\beta_1S_j+{\textstyle\sum_{k=1}^K}\beta_jX_{ij}+\varepsilon_{ij}$$

*Y*_*ij*_ refers to the outcome variable for woman *i* (whether she sought healthcare: yes vs. no), with *j* category of explanatory variables. S refers to the categorical variable of perceived severity of illness, with the category of women with no knowledge of signs or symptoms as the reference. *β*_1_ provides the odds ratio (OR) for each category of knowledge of perceived severity of illness, except the reference category. *X*_*ij*_ denotes a vector of explanatory variables, and $k$ refers to the number of explanatory variables. *β*_*0*_ stands for the intercept term, and β_*j*_ refers to the OR for each category of explanatory variables, except the reference category of each variable.ε_ij_ refers to the error term. Because data were collected at the household level, we added a random cluster effect in our model estimates to take the clustering effects of data at the household level into account, and to adjust standard errors for the ORs and 95%CIs. The GLM statistical model was fitted on each of the three outcomes, separately, and adjusted for the knowledge variable and sociodemographic characteristics.

## Ethics

Secondary data were used in this study, and it was not required to obtain ethics approval.

## Results

Table 1 shows that there were 9,190 ever-married women, aged 13–49 years, who gave birth in the past 2 years. Sixty two percent of women were 13–29 years old, and majority of women (77%) lived in rural. Most women (78%) lacked formal education, and only one in 10 women knew 3–5 danger signs or symptoms during pregnancy. Majority of women (87%) gave birth more than once in their life span. For nearly two thirds of women their husbands (44%) and their in-laws and relatives (21%) decided where the women would give birth. Three out of five women (59%) had their latest deliveries conducted by healthcare professionals. Access of women to radio, TV, and the internet was 28%, 34%, and 3%, respectively.

**Table 1:** Characteristics of women by sociodemographic factors, and knowledge of severity of illness

		N	(%)
Age group	^a^13-29 years	5695	(62)
	30–39 years	2831	(31)
	40–49 years	664	(7)
Education level	No formal education	7141	(78)
	Primary (1–6 years schooling)	847	(9)
	Intermediate (7–9 years schooling)	557	(6)
	Secondary (10–12 years schooling)	443	(5)
	Higher education	202	(2)
Socioeconomic status	Poorest	1707	(19)
	Poor	1762	(19)
	Middle	1954	(21)
	Rich	1956	(21)
	Richest	1811	(20)
Place of residence	Urban	2096	(23)
	Rural	7094	(77)
Knowledge of severity of illness	Women with no knowledge of sign/symptom	3942	(43)
	Women who knew 1 sign/symptom	2137	(23)
	Women who knew 2 signs/symptoms	2223	(24)
	Women who knew 3–5 signs/symptoms	888	(10)
Multiparity	One delivery	1233	(13)
	≥ 2 deliveries	7957	(87)
Decision that where the woman to deliver	By herself	3180	(35)
	By Husband	4077	(44)
	By mother or mother- or father-in law	1739	(19)
	By friends or relatives	194	(2)
Last delivery was conducted by	Doctor	1172	(12)
	Nurse	134	(2)
	Midwife	4124	(45)
	Non-medical person	3760	(41)
Access to radio (whether the woman listens to radio)	Does not listen to radio	6550	(71)
	Listens almost every day	1047	(12)
	Listens at least once a week	1593	(17)
Access to TV (whether the woman watches TV)	Does not watch TV	6065	(66)
	Watches almost every day	2218	(24)
	Watches at least once a week	907	(10)
Access to internet (whether the woman uses internet)	Does not use internet	8879	(97)
	Uses almost every day	125	(1)
	Uses at least once a week	186	(2)

^a^There were one 13 years, two 14, six 15, twenty-five 16, and fifty-nine 17 years old ever-married women

**Fig. 1:**
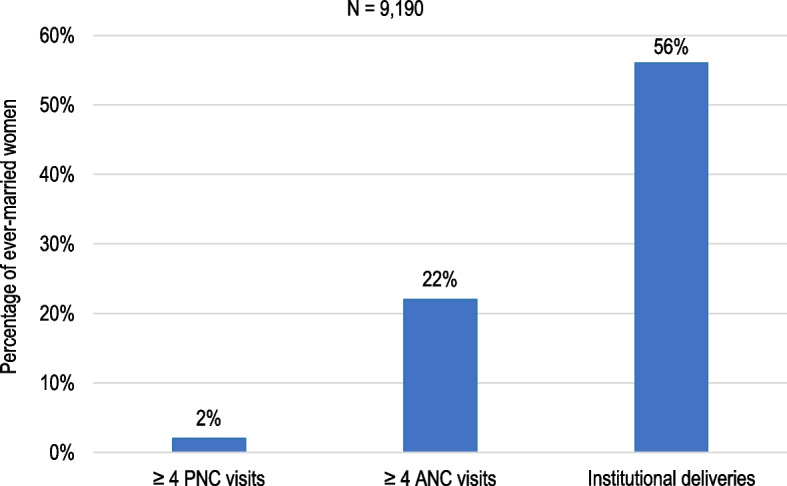
Women who sought pregnancy-related health services

Figure 1 shows that 56% out 9,190 ever-married women sought institutional delivery services; but only 22% and 2% of women sought healthcare for ≥ 4 ANC and ≥ 4 PNC visits, respectively.

**Fig. 2:**
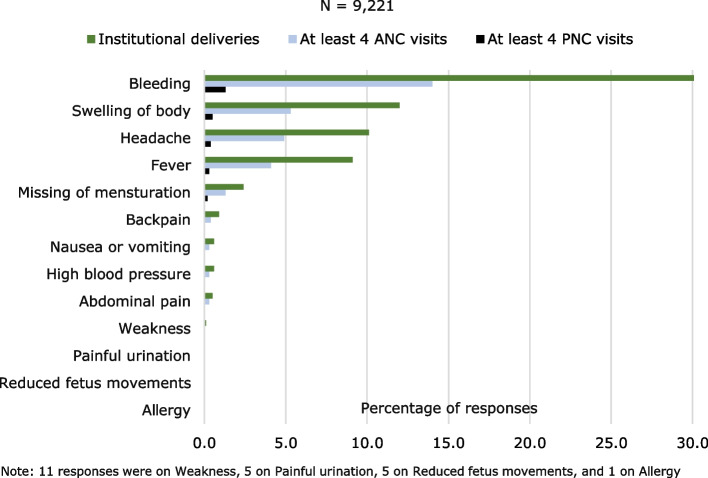
Women’s responses on danger signs or symptoms related to pregnancy or childbirth

Figure 2 shows that 45% of responses provided by women was on bleeding (30% on institutional deliveries, 14% on ≥ 4 ANC, and 1% on ≥ 4 PNC visits), swelling (18%), headache (15%), fever (14%), others (8%).

Table 2 shows that women’s knowledge of danger signs or symptoms during pregnancy is a strong predictor of pregnancy-related healthcare seeking behavior [Odds Ratio (OR) and 95%CI of 1.77(1.54–2.05), 2.28(1.99–2.61), and 2.78 (2.34–3.32) on knowledge of 1, 2, and 3–5 signs or symptoms, respectively] in women with ≥ 4 ANC visits when compared with women who did not know any of the signs or symptoms. In women with ≥ 4 PNC visits, the likelihoods were 1.80(1.12–2.90), 2.22(1.42–3.48), and 3.33(2.00–5.54), respectively, and in women with institutional deliveries, the likelihoods were 1.49(1.32–1.68), 2.02(1.78–2.28), and 2.34(1.95–2.79), respectively. Unadjusted ORs on woman’s knowledge of danger signs and symptoms are presented in Additional file 1. As expected, it was revealed that education is a significant predictor of healthcare seeking in women with ≥ 4 ANC visits [OR 1.36(1.15–1.61), 1.40(1.14–1.71), 1.50(1.21–1.86), 1.53(1.12–2.07) for primary, intermediate, secondary, and higher education level, respectively]. In women with ≥ 4 PNC visits, education at higher levels was significant on healthcare seeking behavior [OR 1.95(1.13–3.36) for secondary, and 2.62(1.37–4.99) for higher education]. Education was a significant predictor of seeking institutional delivery services [OR 1.49(1.25–1.76) for primary, 1.46(1.18–1.82) for intermediate, 1.99(1.54–2.59) for secondary, and 2.52(1.58–4.03) for higher education]. In terms of multiparity, it was less likely that women with ≥ 2 childbirths to seek institutional delivery [OR 0.67(0.58–0.78)] when compared with women who gave birth once. A similar pattern was observed on institutional delivery for women living in rural areas [OR 0.61(0.53–0.71)] when compared with women living in urban areas. As expected, it was found that socioeconomic status is a significant predictor of healthcare seeking. In women with ≥ 4 ANC visits at the highest and second highest quintile of socioeconomic status the likelihood of seeking the services was higher [OR 1.30(1.06–1.59), and 1.53(1.22–1.92, respectively)] when compared with women at the lowest quintile. In comparison to women at the lowest quintile, women from the remaining four quintile of socioeconomic status had higher likelihood of seeking institutional delivery [OR 1.83(1.57–2.13), 2.60(2.23–3.03), 3.24(2.76–3.81), and 5.38(4.36–6.63) from the second lowest to the highest status]. Access to mass media was a significant predictor of seeking healthcare; women who listened to radio daily had higher odds of seeking institutional delivery [OR 1.29(1.10–1.51)] as compared with women who did not listen to radio. Women who watched TV daily had higher odds of seeking ≥ 4 ANC visits and institutional delivery [OR 1.64(1.43–1.88), and 1.50(1.31–1.71, respectively)], and women who watched TV at least once a week had a similar pattern for ≥ 4 ANC visits and institutional delivery [OR 1.27(1.06–1.53, and 1.41(1.19–1.68), respectively]. In women who used the internet at least once a week, the odds of seeking ≥ 4 ANC visits and institutional delivery was significantly higher than women who did not use the internet [OR 1.64(1.21–2.23), and 1.55(1.02–2.37), respectively]. In women whose latest delivery was conducted by a healthcare professional, it was more likely the women would seek ≥ 4 ANC visits [OR 2.93(2.45–3.50), 1.70(1.10–2.63), and 2.56(2.23–2.92) for medical doctors, nurses, and midwives, respectively] when compared with women whose latest delivery was conducted by a non-medical person. The same was true for ≥ 4 PNC visits [OR 4.95(2.62–9.35), 7.19(2.60–19.93), and 4.25(2.39–7.56) for medical doctors, nurses, and midwives, respectively]. Compared with women’s decision, the decision of husband, in-laws, and friends and relatives for women where to give birth was significant for institutional delivery [OR 3.53(3.16–3.95), 3.20(2.78–3.68), and 2.76(1.98–3.86), respectively]. We did not find that age is a strong predictor of healthcare seeking behaviour for maternal health services.

**Table 2:** Likelihood of seeking at least 4 ANC and 4 PNC visits, or having institutional delivery

Predictor	≥ ANC visits	≥ PNC visits	Institutional delivery
^b^Women’s knowledge of danger signs/symptoms			
No knowledge	Ref	Ref	Ref
1 danger sign/symptom	1.77** (1.54–2.05)	1.80** (1.12–2.90)	1.49** (1.32–1.68)
2 danger signs/symptoms	2.28** (1.99–2.61)	2.22** (1.42–3.48)	2.02** (1.78–2.28)
3–5 danger signs/symptoms	2.78** (2.34–3.32)	3.33** (2.00–5.54)	2.34** (1.95–2.79)
Age group			
13-29 years	Ref	Ref	Ref
30–39 years	1.07 (0.95–1.21)	0.95 (0.65–1.40)	1.06 (0.96–1.18)
40–49 years	1.00 (0.81–1.24)	0.57 (0.24–1.33)	1.12 (0.92–1.36)
Education level			
No formal education	Ref	Ref	Ref
Primary (1–6 years schooling) education	1.36** (1.15–1.61)	1.50 (0.91–2.47)	1.49** (1.25–1.76)
Intermediate (7–9 years schooling)	1.40** (1.14–1.71)	1.35 (0.76–2.38)	1.46** (1.18–1.82)
Secondary (10–12 years schooling)	1.50** (1.21–1.86)	1.95* (1.13–3.36)	1.99** (1.54–2.59)
Higher education or university	1.53** (1.12–2.07)	2.62** (1.37–4.99)	2.52** (1.58–4.03)
Multiparity			
One delivery	Ref	Ref	Ref
≥ 2 deliveries	0.86 (0.73–1.00)	0.73 (0.48–1.11)	0.67** (0.58–0.78)
Place of residence			
Urban	Ref	Ref	Ref
Rural	1.14 (0.98–1.32)	0.92 (0.60–1.41)	0.61** (0.53–0.71)
Wealth status			
Poorest	Ref	Ref	Ref
Poor	1.12 (0.92–1.37)	0.97 (0.45–2.08)	1.83** (1.57–2.13)
Middle	1.19 (0.98–1.45)	1.50 (0.75–2.99)	2.60** (2.23–3.03)
Rich	1.30** (1.06–1.59)	1.13 (0.55–2.33)	3.24** (2.76–3.81)
Richest	1.53** (1.22–1.92)	1.18 (0.54–2.59)	5.38** (4.36–6.63)
Access to radio			
Does not listen to radio	Ref	Ref	Ref
Listens almost every day	0.97 (0.83–1.15)	0.87 (0.54–1.40)	1.29** (1.10–1.51)
Listens at least once a week	0.87 (0.76–1.01)	0.81 (0.52–1.25)	1.03 (0.90–1.17)
Access to TV			
Does not watch TV	Ref	Ref	Ref
Watches almost every day	1.64** (1.43–1.88)	1.47 (0.96–2.25)	1.50** (1.31–1.71)
Watches at least once a week	1.27** (1.06–1.53)	1.45 (0.85–2.46)	1.41** (1.19–1.68)
Access to internet			
Does not use internet	Ref	Ref	Ref
Uses almost every day	1.39 (0.95–2.03)	1.90 (0.90–4.01)	1.26 (0.73–2.17)
Uses at least once a week	1.64** (1.21–2.23)	1.08 (0.52–2.26)	1.55* (1.02–2.37)
^a^Latest delivery conducted by			
Non-medical person	Ref	Ref	
Doctor	2.93** (2.45–3.50)	4.95** (2.62–9.35)	
Nurse	1.70** (1.10–2.63)	7.19** (2.60–19.93)	
Midwife	2.56** (2.23–2.92)	4.25** (2.39–7.56)	
Decision that where the woman to deliver			
By herself			Ref
By Husband			3.53** (3.16–3.95)
By mother or mother- or father-in law			3.20** (2.78–3.68)
By friends or relatives			2.76** (1.98–3.86)

^*^ *p*-value < 0.05, ** *p*-value < 0.01. ^a^This variable had collinearity with institutional delivery

^b^Bivariable analysis (unadjusted OR) on knowledge of danger signs or symptoms is provided in the supplementary material

**Fig. 3:**
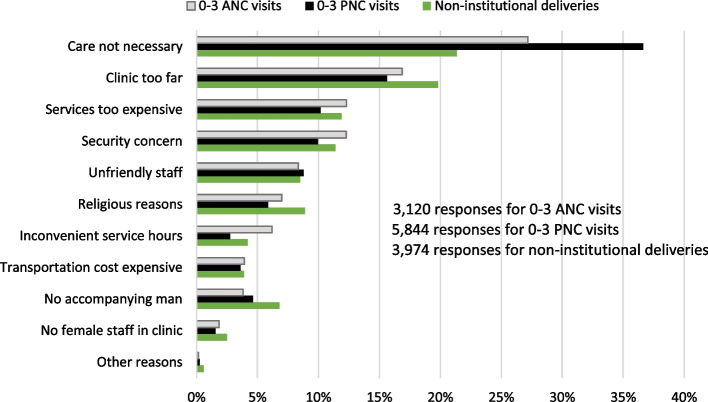
Reasons for not seeking at least 4 ANC, PNC visits, or institutional deliveries

Figure 3 shows responses by women who did not seek ≥ 4 ANC, ≥ 4 PNC visits, or institutional deliveries. It shows that one category of responses (care not necessary) was on perceived susceptibility to illness and the remaining ten categories were on perceived barriers. In women with 0—3 ANC visits, 27% thought that care was not necessary. Other responses were clinic too far (17%), too expensive services (12%), security concern (12%), unfriendly staff (8%), religious reasons (7%), inconvenient service hours (6%), transportation cost expensive (4%), not having an accompanying man (4%), no female staff (2%).

In women with 0—3 PNC visits 37% thought that care was not necessary. Other responses were clinic too far (16%), too expensive services (10%), security concern (10%), unfriendly staff (9%), religious reasons (6%), inconvenient service hours (3%), transportation cost expensive (4%), not having an accompanying man (5%), no female staff (2%).

In women who did not seek institutional deliveries 22% said that care was not necessary. Other responses were clinic too far (20%), too expensive services (12%), security concern (12%), unfriendly staff (9%), religious reasons (9%), inconvenient service hours (4%), transportation cost expensive (4%), not having an accompanying man (7%), no female staff (3%).

## Discussion

In this study of 9,190 ever-married women, we found that only 2% of women sought healthcare for at least 4 PNC visits, 22% for at least 4 ANC visits, and 56% of women sought care to give birth at health facilities. It was revealed that women's knowledge of perceived severity of illness during pregnancy increases the likelihood of healthcare seeking for at least 4 ANC, PNC visits, or institutional deliveries. It was found that except the woman’s age, other sociodemographic characteristics of women were strong predictors of healthcare seeking for at least 4 ANC, PNC visits, or institutional deliveries. The characteristics were women’s education level, socioeconomic status, access to media (radio, TV, internet), husband’s decision, in-laws’ and relatives’ decision for women where to give birth, residential area (urban vs. rural), multiparity, and access of women to healthcare professionals for childbirth services. Our findings suggest that the decisions of husband, in-laws, and friends and relatives for women where to give birth are more likely to have institutional delivery than decisions by women themselves. Although other studies found the importance and effectiveness of women’s decision on institutional delivery, our study tells us the importance of husband, in-laws, and friends and relatives’ knowledge of institutional delivery, especially when women’s decision making for place of delivery remains limited.

Our results support the augmentation of women education and media access with the use of maternal health services. In addition to women’s formal education, research shows that women’s participation in community groups is an effective approach to promote women’s and children’s health in LMICs [42]. In a systematic review that studied women’s groups participatory learning at community level and the use of maternal health services, it was found that women’s groups learning resulted in increases in the use of institutional deliveries, ANC visits, and immediate PNC checks and follow-up visits. There is an argument on the improved links between community-based initiatives and health facility-based interventions, including the quality of clinical care [43]. For this to happen, more collective activities would be needed, requiring moving beyond women’s groups learning as the main agent of change [42].

Our finding on the coverage of ANC usage is consistent with the findings reported from LMICs [13, 17]. According to recent studies that used multi-country data, Afghanistan has one of the lowest coverage of ANC utilization compared to several other LMICs [13, 17, 44]. In a study that analyzed national representative population-based data from 54 LMICs, it was found that Afghanistan, based on its 2015 DHS data, placed at the lowest quartile in terms of number of ANC visits [13]. Not only the ANC coverage is very low in Afghanistan, the quality of ANC services is also poor [17]. Quality of ANC services, defined as a content qualified ANC indicator, was measured in a multi-country study from national representative data from 63 LMICs, and it was revealed that it ranged from 3.5 (out of 10) in Afghanistan to 9.3 in Cuba [17]. Our findings, which are even before the collapse of the Afghan government to the Taliban in August 2021, show that Afghanistan has a lowest coverage of at least 4 PNC visits, compared with findings from other LMICs [19–21, 45]. The coverage of at least 3 PNC attendance was 62% in a study [45], and the utilization of early PNC visits ranged from 11 to 23% to 50% in three studies from LMICs [19–21]. Our finding on the coverage of institutional deliveries is consistent with those reported previously from Afghanistan [31, 33, 35]. In one study the deliveries conducted by SBA was reported as 59% [35], and in a previous study the coverage of institutional delivery was reported as 38% [33], and in a recent study, that used data from a health facility in Kandahar city, the coverage of institutional delivery was reported as 72%. The difference of 16% (between 72 and 56% in our study) seems to be due to fact that data for the recent study in Afghanistan come from an urban clinic [31].

Our findings on women’s knowledge support those previously reported from LMICs. Previous studies from LMICs reported strong associations between women’s knowledge of danger signs or symptoms during pregnancy, childbirth, or postpartum and utilization of maternal healthcare [46, 47]. A study from Laos found that women’s knowledge on obstetric care was associated with the use of reproductive healthcare such as ANC visits, tetanus toxoid (TT) vaccination, and SBA deliveries [46]. In a study in Nigeria, it was reported that the likelihood of healthcare seeking was significantly lower in women who had poor knowledge of the causes of maternal deaths than women with good knowledge of the causes of maternal deaths [47]. A study from Zimbabwe identified that only women who had experience of complications during pregnancy were able to describe danger signs related to pregnancy and childbirth, such as bleeding, high blood pressure, uncontrollable vomiting, stomach pain, diarrhoea, premature labour, breech presentation, and amniotic fluid leakage [48]. In a study in Bangladesh, it was found that 42% of the respondent women knew about swelling of the feet, 36% were aware of seizures, 26% knew about headache, and 25% knew about bleeding as warning signs related to pregnancy [49]. Another study from a national survey in Bangladesh revealed that 56% of women cited tetanus, 49% cited prolonged labour, 38% named retained placenta, and 25% mentioned malpresentation of the fetus as potential life-threatening conditions [50]. Eighty nine percent of women were able to name one or more of the conditions related to pregnancy or childbirth, and 42% could name three or more of such conditions [50].

Several studies from LMICs examined determinants of women's healthcare seeking behavior for reproductive health services, using the Health Belief Model (HBM) [51]. The HBM includes six constructs: perceived susceptibility, severity, benefits, barriers, self-efficacy, and action cues [51]. The demographic, socioeconomic, and cultural variables (e.g., age, race, ethnicity, education, income) can influence behavior [51]. In a study, which employed the HBM’s constructs of perceived risk, self-efficacy, and cues to action, the likelihood of SBA deliveries among Ghanaian women was examined [52]. The authors reported that women whose deliveries were conducted by SBAs believed that they were prone to complications during childbirth, had high perceived self-efficacy to overcome barriers to deliver with SBAs, and were well-prepared to deliver with SBAs. In contrast, women who did not deliver with SBAs were less likely to believe in susceptibility to complications, had low perceived self-efficacy to overcome barriers, and were not prepared to deliver with SBAs in advance [52]. In our study it was found that women whose latest delivery was conducted by SBAs were more likely to seek ≥ 4 ANC, ≥ 4 PNC visits when compared with women whose latest delivery was not conducted by SBAs, and this suggests that these women were awareness of their susceptibility to complications and were preparing in advance during pregnancy to deliver with SBAs. Using the HBM, a study in Nigeria found that women’s perceived susceptibility to complications during pregnancy and childbirth influences healthcare seeking behavior [53]. Acquisition of knowledge of causes and signs of pre-eclampsia, the quality of ANC services, and supportive discussions with families were influencing factors [53]. In another study in Ethiopia, it was revealed that perceived susceptibility to and perceived severity of delivery-related complications were positively correlated with women’s intention to deliver at a health facility [54]. In our study we found that women’s knowledge of danger signs or symptoms during pregnancy influences the usage of pregnancy-related services, including ≥ 4 ANC visits and institutional delivery. In the study in Ethiopia, it found that intention to deliver at a health facility was negatively associated with women’s perceived barriers to accessing the facility [54]. Similar findings on perceived distance and initiating of timely ANC visits were reported from a multi-country analysis of DHS in 39 African countries [12]. In our study, perceived barriers according to the women who did not use the recommended number of 4 ANC, PNC visits, and institutional deliveries were, distance to a clinic, expensive health services, security concerns, unfriendly staff, religious reasons, inconvenient service hours, expensive transportation cost, lack of female staff in clinic, and no accompanying man. Our findings on perceived barriers are consistent with the findings from previous studies in Afghanistan [32, 39].

Our findings on the relationship of sociodemographic characteristics of women, except woman’s age, and healthcare seeking for maternal health services are consistent with those from the latest studies from LMICs [12, 44]. In a recent meta-analysis, using DHS data from 29 LMICs, it was found that place of residence [OR 2.04(1.62–2.57)], woman’s age [OR 1.26(1.11–1.44)], education level [OR 2.81(2.35–3.35)], wealth status [OR 2.72(2.20–3.35)], birth order [OR 1.72(1.39–2.14)], and access to media [OR 2.47(2.10–2.91)] had significant influence on woman’s decision to attend ANC visits [44]. Similar findings on the initiation of ANC visits within the 12 weeks of pregnancy were reported from a multi-country analysis of DHS data from 39 African countries [12]. In another recent study, it was reported that women with no knowledge of pregnancy complications were less likely to utilize ANC services within the first trimester (OR 0.76, p < 0.01), achieve the WHO recommended minimum 8 ANC visits (OR 0.66, p < 0.01), and deliver at a health facility (OR 0.77, *p* < 0.10) [55]. A recent study from Afghanistan found that women with lack of knowledge on when to start ANC attendance were more likely to start the services later [37].

An unexpected finding in our study was the insignificant relationship of women age with seeking pregnancy-related health services. Previous and recent studies from LMICs, including Afghanistan, provide evidence that age is a significant influencer of the utilization of pregnancy-related health services by pregnancy women [12, 29, 31, 33, 35, 36, 44]. In light of the evidence provided by previous and recent studies, based on our findings we cannot state that age may not be a strong predictor of seeking healthcare for pregnancy-related services, even if two recent studies that employed data from a health facility in Afghanistan did not find a strong relationship between woman’s age and use of ANC services [34, 37].

The strengths of our study include the use of data from a nationally representative sample, clear instructions to surveyors to minimize bias, the dose–response relationship between women’s knowledge of perceived severity of illness and healthcare seeking for at least 4 ANC, PNC visits and institutional deliveries, and the presenting of findings on the relationship of women’s sociodemographic characteristics and utilization of ≥ 4 ANC, ≥ 4 PNC visits, and institutional deliveries in one paper. The use of data from this national survey permits generalizability of our findings at the national level, and the use of questionnaire in AHS2018, which is similar to the questionnaires applied in the demographic health surveys (DHS), allows comparison of our findings to those from other LMICs when a study similar to ours, is conducted in the LMIC(s). In addition, the relatively large sample size in our study may increase the statistical power of the findings. The surveyors were local educated individuals who were recruited for the AHS2018. The surveyors were trained on the household survey, and clear instructions alongside the survey field manual were provided to them on how to ask the questions, using the questionnaire, and conduct interviews in the selected households. The instruction of “no replacement of selected households to other households” would minimize the chance of selection bias. Moreover, the written instruction, as a footnote under the question on women’s knowledge of perceived severity of illness, would restrain surveyors from providing any hints to the woman during the interview. This would minimize the change of introducing bias to the responses provided by the interviewee women. Furthermore, our findings on the dose–response relationship between women’s knowledge and healthcare seeking would enable us to examine the associations between different levels of knowledge (e.g., knowing 1 sign or symptom, knowing 2 signs or symptoms, knowing 3–5 signs or symptoms) and the likelihood of healthcare seeking for maternal healthcare. This suggests that the more a woman knows about reproductive health the greater would be the likelihood she would seek healthcare. Presentation of our findings on the relationship of women’s sociodemographic characteristics and healthcare seeking for ≥ 4 ANC, ≥ 4 PNC visits, and institutional deliveries in one paper has the advantage that a reader of the paper can compare the consistency or inconsistency on the effect of each characteristic on the utilization of ≥ 4 ANC, ≥ 4 PNC visits, and institutional deliveries, using the latest countrywide data available from Afghanistan.

Our study's limitations include the use of secondary data, the lack of a theoretical cognitive model (e.g., HBM), social desirability, and use of old data. The use of secondary data, which did not include questions specifically designed based on a theoretical cognitive model, is a limitation. In studies that examine perceptions of users of health services in relation to their intentions to change behavior, theoretical cognitive models are applied [51]. These models include different components of user perceptions (e.g., perceived susceptibility, perceived severity, perceived benefits, perceived barriers, perceived self-efficacy). In our study, however, we did not have the resources, including funding, to undertake the study using a theoretical cognitive framework, which would have guided the development of questions on different components of women’s perceptions and their relationships with seeking healthcare for ANC, PNC visits, and institutional deliveries. Moreover, the phrase “the need for you to seek urgent care” in the question on women’s knowledge can be interpreted both perceived severity and perceived susceptibility. The phrase may remind the woman to name signs or symptoms that would prompt her to seek healthcare immediately. She may have perceived that she was susceptible to the consequences of the condition(s); therefore, urgent care must have been sought. The phrase may also be interpreted as perceived severity of illness, because the phrase may have probed the woman to name the sign(s) or symptom(s) that indicate the seriousness of the condition(s) for which the woman would have to seek urgent care. Another limitation concerns the social desirability issue, potentially emerging from self-reported data. Because women reported the number of ANC, PNC visits, and institutional deliveries, it is possible that the women may have over-reported the number of ANC, PNC visits or their deliveries at health facilities or hospitals. Yet another limitation concerns the use of data from AHS2018, which was conducted five years ago. Over the past five years, healthcare seeking may have changed in Afghanistan. The impact of COVID-19 pandemic on maternal healthcare is another concern that our study cannot address. There is evidence that COVID-19 pandemic caused disruptions in the provision and utilization of maternal health services in LMICs [56, 57]. Since early 2020 that COVID-19 was officially announced by WHO as a pandemic, the healthcare system in Afghanistan has encountered serious challenges to contain the virus and to deal with a high number of patients needing care for COVID-19 [58–60]. Challenges such as the insufficiency of donor funds, unstable political situation, inadequate equipment and facilities, insufficient health professionals, shortage of the COVID-19 vaccines, illiteracy of people, and poverty are threatening the lives of millions of people in Afghanistan [59, 60]. The situation has worsened after the political and military advance of the Taliban in August 2021, and the reduction in healthcare capacity, and existence of humanitarian crisis [7, 59]. Despite the evidence on the possibility of another wave of COVID-19 in the country, the Taliban’s de facto authority do not seem to have a clear plan to combat against the pandemic when it occurs [60]. One solution is to integrate the COVID-19 services in the existing package of healthcare services, which may enable the healthcare system to combat the infection in the middle- and long-term [60]. However, this integration may put further pressure on the already ill-prepared healthcare system with the possibility of shifting resources from maternal and child health care to COVID-19 services. Our findings from the AHS2018 data are best suitable to a relatively normal situation of Afghanistan where the healthcare system is (reasonably) adequately funded, and the system is not under pressure from the epidemic of a highly contagious infectious disease, and the country is not in humanitarian crisis. This is despite the fact that our findings on sociodemographic characteristics of women and maternal healthcare seeking, overall, are consistent with the findings from recent studies in 2022 from Afghanistan [31, 34, 37]. These three recent studies used data from a health facility in Kandahar city. Since August 2021, considerable migration of more educated women out of Afghanistan and humanitarian crisis emerging from continued violation of human and woman’s rights may have serious consequences to maternal and child healthcare. Even if in some parts of the country, especially in rural areas, security concern may not be a major issue now; however, the costs associated with healthcare seeking (e.g., transportation) or lack of an accompanying male or lack of female staff in clinics may pose major obstacles to accessing maternal health services in Afghanistan. With the Taliban’s decision to ban women from working with humanitarian organizations it is likely that women’s access to maternal health services would further decrease. The Taliban’s women employment ban has resulted in many international organizations being forced to restrict their work in the country, which raises further concerns about the accessibility of maternal health services for pregnant women in Afghanistan [61]. This has the potential to further entrench existing gender inequalities and slow efforts to create health care systems that are inclusive and gender responsive.

Moreover, access to mass media may be restricted now than five years ago, and the media may not have the freedom to air health promotion messages to the public without the Taliban-led government’s approval. This study found that women who used the internet at least once a week had higher odds of seeking four or more ANC visits and delivering in healthcare facilities. With the current ban on women's employment, many women may lose their economic independence and they and their families may not be able to afford internet access, potentially leading to a decline in the knowledge level of current and future generations of women. Equally, if not more, important is the concern on early marriage of girls, as with the Taliban's strict gender rules and disregard for women's rights it is likely that the risk of early marriage for girls would increase, leaving them vulnerable if they lack knowledge about maternal health [62].

Our findings have the potential to influence health policies and interventions to promote healthcare seeking behavior for maternal health services. The very low prevalence of at least 4 PNC, at least 4 ANC visits, and the 44% non-institutional deliveries may influence the development and revision of health policies and interventions related to reproductive healthcare for women. This is despite the fact that recently WHO has recommended the minimum number of ANC to increase from 4 to 8 visits during the span of a normal pregnancy [5], and some studies in LMICs have used the updated WHO’s recommendation in their work [55]. For our study it means that if you used the minimum recommended 8 ANC visits, the coverage of ANC visits may have been much lower than the 22% which we found in this study. Our findings suggest that the higher the level of women’s knowledge of perceived severity the greater is the likelihood that women would seek healthcare for maternal health services. Our findings that nearly one thirds of women, who did not seek healthcare, thought that care was not necessary emphasizes the need to focus on providing health education to women on topics related to reproductive health, especially those health messages that increase women’s knowledge of perceived susceptibility to and perceived severity of illnesses. Considering the Taliban’s strict rule on women’s education and employment, it is advisable that health education on promoting community’s knowledge, particularly women’s knowledge, of perceived severity of and susceptibility to illness could be conducted at public health facilities and aired through mass media under the leadership of the Ministry of Public Health and with coordination of UN agencies.

Future research may study associations between distance or travel time to health facilities and healthcare seeking, including delays in commencing the first ANC visits, quality of ANC services (e.g., type of services provided to a pregnant woman during an ANC visit), and initiation of timely PNC check-up and follow-up visits. Another venue to explore may be the cost of using maternal health services, including transportation costs and their relationships with healthcare seeking. The consequences of women’s ban from secondary and higher education as well as women’s ban from working with humanitarian organizations on accessibility of maternal health services to pregnant women would be another important venue to examine. Future research could also investigate women's perceptions of the severity, susceptibility, benefits, barriers, and self-efficacy of these issues in relation to their use of maternal reproductive health services.

## Conclusion

Health policy and interventions are needed to promote women’s knowledge of perceived susceptibility to, and severity of illness related to reproductive health and utilization of maternal health services. Community- and clinic-based interventions need to be implemented to provide health education on danger signs or symptoms during pregnancy, childbirth, and postpartum period. Health education messages need to be disseminated through mass media, and health clinics and hospitals, targeting women, especially young women. Determinants of poor healthcare seeking (e.g., poor access to health facilities, unfriendly staff, shortage of female staff, transportation costs, and expensive health services) need to be addressed.

Women education in the country, with at least primary education in rural areas, as well as improving women’s access to media are recommended, considering that educated women may have a higher change of exposure to information, including that of maternal and child healthcare, and have a greater decision-making power on their own and on their children’s health. These objectives, however, cannot be easily achieved in the current humanitarian crisis in Afghanistan where the Taliban’s decision to ban women from secondary and higher education and from working with humanitarian organizations has further worsened the humanitarian crisis and human suffering.

## Supplementary Information

**Additional file 1.** 

## Data Availability

Data supporting the findings reported in the manuscript can be requested from the Ministry of Public Health of Afghanistan (Contact person: Dr Saeedzai atasayedzai@gmail.com).
