# Supplementary material for: Predicting maternal healthcare seeking behaviour in Afghanistan: exploring sociodemographic factors and women’s knowledge of severity of illness
Source: BMC Pregnancy Childbirth. 2023 Aug 2;23:561. doi: 10.1186/s12884-023-05750-y (PMC10398983; doi:10.1186/s12884-023-05750-y)
Supplement: Supplementary file 1 — Additional file 1. [file 12884_2023_5750_MOESM1_ESM.pdf]

Appendix 1. Likelihood of healthcare seeking for at least 4 ANC, at least 4 PNC visits, or institutional deliveries

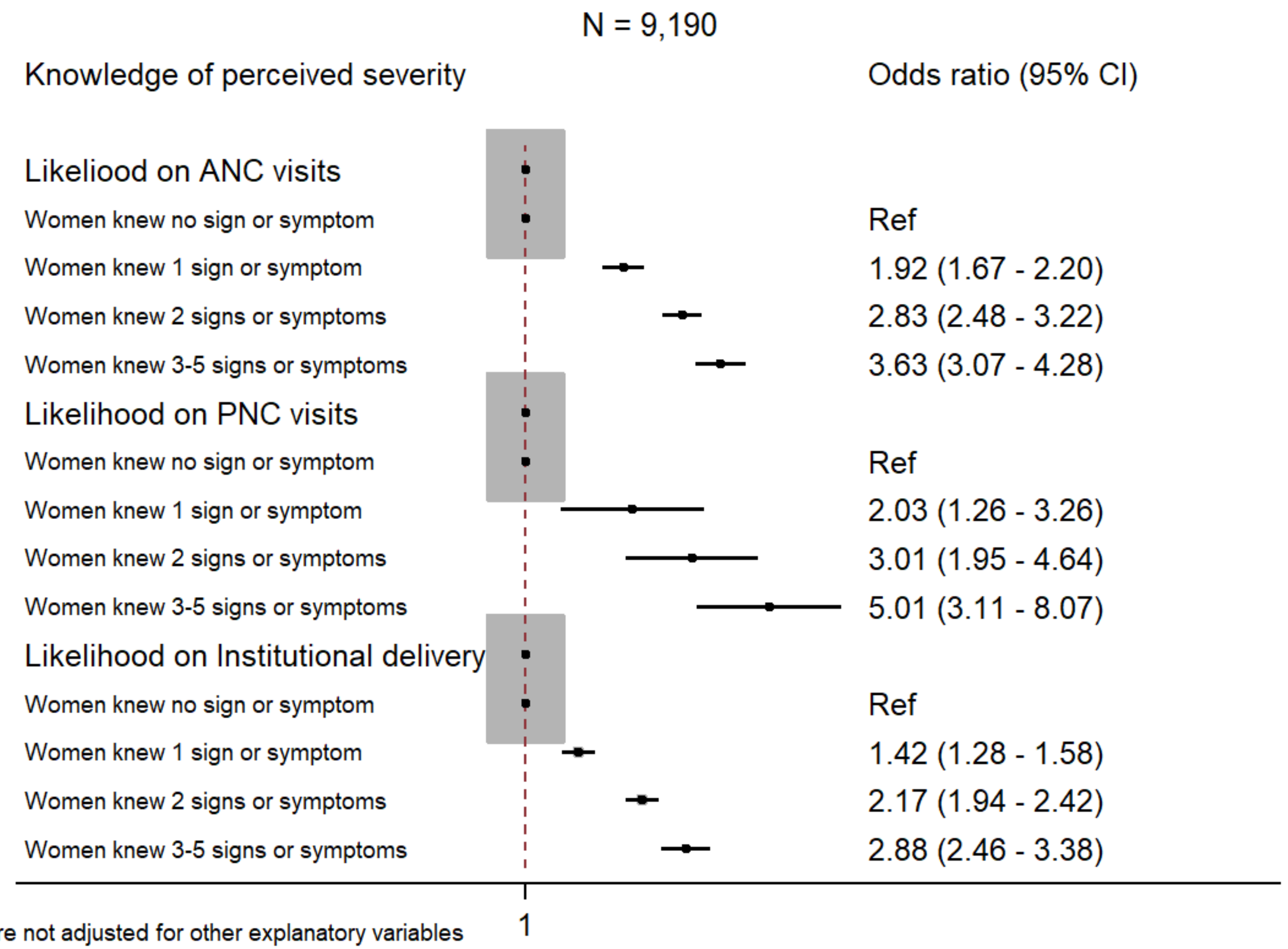

Note: Odds ratios were not adjusted for other explanatory variables

Odds radios were statistically significant (p-value < 0.001)
